# Medals vs. Patents

**Published:** 1865-01

**Authors:** 


Medals *vs.* Patents.—We are pleased to see that the Mississippi Valley Dental Association in a laudable and earnest effort to stimulate the inventive genius of their professional brethren, have offered a silver medal for the most valuable discovery, improvement or invention pertaining to dentistry, during the present year. The proposition is not confined to those engaged in the dental profession, but is extended to everybody, whether dentist, physician or mechanic. Information in regard to it may be obtained of Dr. A. S. Talbert, Lexington, Kentucky, or Dr. Shadoan, box 2079, Cincinnati, Ohio.

This is not a new thing with our friends of the Far West, who are ever moving in the right direction, just at the right time. Dental patents are being frowned upon; societies are being formed for mutual protection against the claims of patentees; a rebellion is being commenced against a class in the profession whose services are almost indispensable. It is to be regretted that our inventors have sought remuneration for their trouble by means of letters patent, and more to be lamented that they continue to do so. It is not to be denied that they have the right, and that it is a just course, but it should be evident to them by this time, that it is not expedient. With the mass it seems to be the determination not to patronize or encourage any one or anything savoring of *patent*, except in a *quiet way*, and we regret to say that that quiet way adds nothing to the yearly income of patentees. Many of the latter have fled for protection and patronage to dental societies, hoping in that way to push their respective improvements through; but after fighting the battle long and ardently, have been compelled to fold their arms and patiently wait for something to "*turn up*" in their favor, or else submit to the force of circumstances, compelled to see the wolves devour their substance.

Improvements and inventions are very often the work of years, with attendant anxiety, expense and care. Idea upon idea, each in its turn, is taken hold of and experimented upon; if answering the purpose, it is taken advantage of, and if not it is cast aside. Little by little, sometimes by accident, sometimes by design, the end is attained, and the toiler expects in some way to be remunerated by those who are benefitted by it. But, unfortunately for him, it is too often regarded as common property, and if he be unselfish and communicative, it is taken from him and others receive the reward. He may die unhonored and unrequited, and his widow and children suffer in poverty. Circumstances may occasionally occur which revive an interest in his memory and their welfare, and to a certain extent justice is done; but this is generally through friends and influence.

It is meet and right that inventors should either be protected by government or receive honorable acknowledgement and reward in the way of a medal or an amount of money; and ample inducements should be offered them to accept the latter. Accepting it, and placing the material for improvement itself in the market, the amount realized, we have not the least doubt, would exceed anything that might be derived from a patent right. For a time the Rubber Company would not allow its rubber, manufactured especially for dentists' use, to be sold to any one but licensees; other dentists received their supply from another source. The sale being large, and the company seeing its mistake, is now willing to have its rubber sold to any one and every one; finding, no doubt, that its income from the sales is many times larger than that from licenses. This should be a useful hint to some who are at present endeavoring to sell their material under cover of a license; and to others, who with unsustained patent rights, but unwilling to acknowledge it, are thus retarding the sale of their goods by the hundred of dollars worth per year, without any corresponding benefit.

Since letters patent have proved to be not only unpopular with dentists, but in the majority of instances failures, the offer of the Mississippi Valley Dental Association is both sensible and opportune, and bids fair to generate a better feeling toward our inventors. Rewards should therefore be offered by every society for improvements of value; and every dentist should consider it his duty to give his mite toward recompensing the originators of them. This is a just and practicable way of discouraging any embarkation in the way of patent rights. A medal or an amount of money should be preferred to lawsuits and the ill-will of professional brethren. It would be better to listen to little flattering speeches, though a great deal be "*soft soap*," than to be compelled to submit to the taunts and slurs of the unthinking and uncharitable.—*Dental Quarterly.*

